# HspB1, HspB5 and HspB4 in Human Cancers: Potent Oncogenic Role of Some of Their Client Proteins

**DOI:** 10.3390/cancers6010333

**Published:** 2014-02-07

**Authors:** André-Patrick Arrigo, Benjamin Gibert

**Affiliations:** Apoptosis, Cancer and Development Laboratory, Lyon Cancer Research Center, INSERM U1052-CNRS UMR5286, Claude Bernard University Lyon 1, Lyon 69008, France; E-Mail: benjamin.gibert@lyon.unicancer.fr

**Keywords:** human small Hsps, HspB1, HspB5, HspB4, Hsp27, alphaA-crystallin, alphaB-crystallin, clients, cancer

## Abstract

Human small heat shock proteins are molecular chaperones that regulate fundamental cellular processes in normal unstressed cells as well as in many cancer cells where they are over-expressed. These proteins are characterized by cell physiology dependent changes in their oligomerization and phosphorylation status. These structural changes allow them to interact with many different client proteins that subsequently display modified activity and/or half-life. Nowdays, the protein interactomes of small Hsps are under intense investigations and will represent, when completed, key parameters to elaborate therapeutic strategies aimed at modulating the functions of these chaperones. Here, we have analyzed the potential pro-cancerous roles of several client proteins that have been described so far to interact with HspB1 (Hsp27) and its close members HspB5 (αB-crystallin) and HspB4 (αA-crystallin).

## 1. Introduction

The literature is filled with reports describing the overexpression of heat shock proteins (Hsps) in many types of cancer cells [1,2]. The phenomenon is probably linked to the drastic changes in protein homeostasis resulting of the accumulation of mutated proteins in cancer cells [2,3,4]. Hsps have been known for a long time as being able to prevent protein aggregation by binding polypeptide clients destabilized during cellular stress, such as heat shock. Among Hsps, the ATP-independent molecular chaperones HspB1, HspB5 and HspB4 belong to the group of small Hsps characterized by a common alpha-crystallin domain in their *C*-terminal part. Recently, small Hsps have been described to have an incredible number of crucial roles in normal unstressed cells as well as in pathological cells where they are usually expressed to high levels. These proteins are now considered as important therapeutic targets, particularly in cancer pathologies [5,6]. Indeed, they display anti-apoptotic and tumorogenic properties and trigger host anti-cancer response inhibition, such as senescence [7,8,9,10,11,12,13]. This results in aggressive cancer cell growth [14,15,16], metastasis formation and dissemination [12,17,18,19] and poor prognosis [2,20,21]. We have proposed that the large panel of cellular functions of small Hsps resulted of their interaction with numerous client proteins whose activity and/or half-life was modified by these chaperones [22,23,24,25]. Small Hsps protein interactomes appear therefore as fundamental parameters to consider when studying the complex activities of these proteins. Here, we have reviewed the pro-oncogenic client proteins interacting with HspB1, HspB5 or HspB4 that can play crucial roles in human cancer pathologies.

## 2. Small Hsps in Human Cancer Pathologies

### 2.1. HspB1

Consequently of the deleterous resistance and enhanced aggressivity brought to cancer cells by HspB1, a high level of expression of this molecular chaperone usually results in poor clinical outcome in the case of gastric, uterine, breast, prostate, ovarian, kidney and head/neck cancers as well as in tumors from the urinary and nervous systems [2,20,21,26,27]. HspB1 is also deeply involved in the invasion of tumor cells into surrounding tissues and formation of metastatic colonies [12,17,18,19,28,29]. Moreover, high levels of HspB1 expression affect tumor susceptibility to adjuvant cancer treatments, including chemotherapy, hyperthermia and radiation therapies [19]. Many client proteins are targeted by HspB1 to promote resistance to cell death and malignant phenotype [23,24,25]. Of particular interest, elevated levels of HspB1 have also been detected in several cancer stem cells, such as those from lung and breast cancers [30,31]. In these cells, HspB1 participates in their maintenance since its elimination suppresses epithelial-mesenchymal transition (EMT) signatures linked to snail, vimentin, E-cadherin and NF-κB [31] and induces long-term dormancy [32]. Snail is a well-defined client protein of HspB1 protected against a rapid degradation consequently of its interaction with this chaperone [33]. A relationship between HspB1 expression and the loss of heterozygosity (LOH) has also been detected supporting the hypothesis that this protein could act as a sensor of genetic imbalances (haploinsufficiency) [34]. Indeed, it was observed that in oligodendroglial tumors HspB1 is a marker of LOH of 1p, information that could help to predict the disease prognosis. Another worth noting point relates to HspB1 ability to enhance cancer cells resistance to a large panel of anti-cancer drugs [35,36,37,38,39,40,41,42,43]. For example, in MCF-7 breast cancer cells exposed to doxorubicin, survival cells are those that overexpress HspB1 consequently of the increased activity of the POU4F2/Brn-3b transcription factor [44]. This factor, which is elevated in more than 60% of breast cancers, modifies growth and behavior of cancer cells by regulating the expression of several target genes [45]. Hence, HspB1 inhibition of drug-induced apoptosis by further enhancing HspB1 expression also increases the cellular resistance to many anti-cancer drugs [46]. Contradictory findings have also been reported. For example, in pancreatic cancer cells, HspB1 has been linked with increased sensitivity [47] as well as with increased resistance [48] to gemcitabine. These opposite observations were reconciled by analyzing an essential parameter of HspB1, that is its phosphorylation status [49]. Crucial effect of phosphorylation has also been observed in Her-2/neu positive breast cancers, where serine 78 phosphorylated HspB1 correlates with Her-2/neu status and lymph node positivity [50]. In that respect, we have recently described the major roles played by the dynamic phosphorylation of HspB1 in the regulation of the anti-apoptotic strategies developped by this protein [51]. While RNAi strategies aimed at therapeutically decrease HspB1 level as well as the search for inhibiting drugs are nowdays deeply active, the use of HspB1 in the clinic is still mainly as a prognostic indicator of specific cancer types [52].

### 2.2. HspB5 and HspB4

The major lens structure proteins HspB5 and HspB4 play essential roles in maintaining normal cellular structure and physiology of both ocular and some non-ocular tissues. Mutations in these polypeptides are responsible of various human diseases such as cataract, neural disorders, and cardiovascular diseases. Of great interest was the recent discovery that abnormal over-expression of these molecular chaperones often occurs in tumors [41,53,54]. The first report describing the association of HspB5 and cancer was by studying breast carcinoma progression [55]. It was originaly observed that HspB5 expression was closely associated with advanced tumor grade progression, lymph vesicular invasion and mortality [53,56]. The phenomenon was particularly intense in “triple-negative” breast tumors [55,56,57], consequently HspB5 is now considered as a biomarker in the diagnosis of breast cancers, especially in those with advanced grade [57,58]. Concerning brain cancer, elevated levels of HspB5 were clearly demonstrated to occur in the more aggressive stages of gliomas [59,60]. Similarly, high levels of HspB5 are associated with low survival rate in hepatocellular carcinoma [61], lung adenocarcinoma [62] and prostatic carcinoma [63]. However, exceptions exist since tumorogenesis can occur in absence of HspB5 upregulation [64]. For example, the role of this protein in kidney tumorogenesis is not clear since an increased level of expression of HspB5 generally occurs in low grade tumors [65], a phenomenon which is in deep contrast to that observed in most breast and brain tumors. HspB5 was also reported to be downregulated in anaplastic thyroid carcinomas [66], nasopharyngeal carcinomas [67], cutaneous squamous cell carcinoma of the head and neck with clinical perineural invasion [68], oral cancer [69,70] as well as human ovarian cancer [71] and testicular tumors [63]. How HspB5 expression can be either up or down regulated in some cancers? At least in breast cancers, HspB5 up regulation may be linked to transcriptional activation. Indeed, HspB5 gene promoter contains an Ets1 binding site that can be activated by the oncogenic transcription factor Ets1 [72]. This Ets1-mediated event appears linked to poor survival.

Compared with HspB1, the functions of HspB5 and HspB4 in tumorogenesis are less known. As mentioned above, HspB5 can promote tumorogenesis while the effects mediated by HspB4 are still equivocal, at least in the few studies that have already been performed [26,73]. Interesting observations have nevertheless been made which showed the dual effect of HspB4 that is either up- or down-regulated depending of the tumor type. For example, elevated levels of HspB4 together with HspB5 are observed in many of the lens tumor cells, while γ-crystallin is selectively reduced [74]. Similarly, HspB4 is also detected in retinoblastoma [41] and in eyelids with sebaceous carcinoma [75]. In these tumors, HspB4 is supposed to prevent apoptosis of neoplastic cells and thus promotes tumorogenesis. In contrast, HspB4, which is expressed at moderate level in normal pancreas, is significantly downregulated in different types of pancreatic carcinoma cells where genetically forced expression of this protein revealed that it can act as a negative regulator that blocks cell transformation and retards cell migration [73]. The effect appears to occur through a modulation of ERK MAP kinase activity [54]. Hence, HspB4 and HspB5 appear to function differentially during tumorogenesis. In contrast to HspB1, therapeutic targetings of HspB5 or HspB4 are still in the stage of experimental exploration [5].

## 3. HspB1, HspB5 and HspB4 Clients and Their Potential Role in Cancer Pathologies

**Figure 1:**
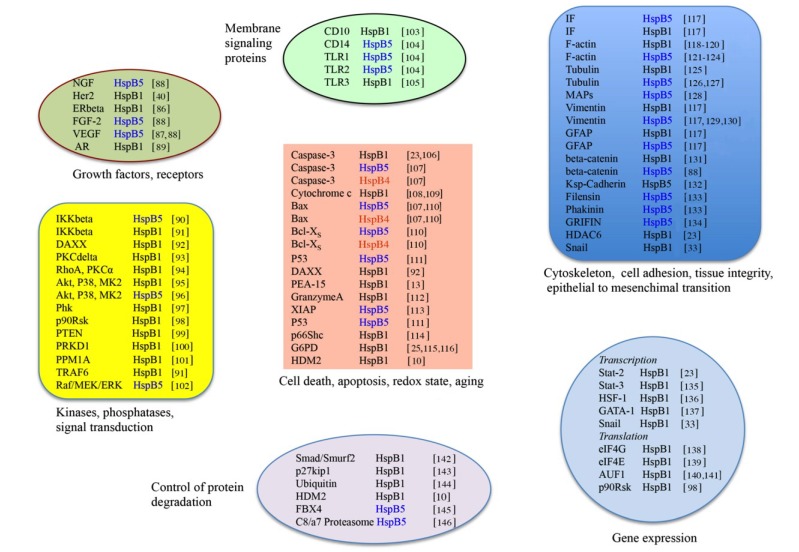
HspB1, HspB5 and HspB4 client proteins and tumor cell growth. The client proteins are listed in function of their tumor-prone activities.

The literature is filled with reports describing the incredible number of roles played by small Hsps, particularly HspB1 and HspB5 in cancer cells. How can these proteins have such a vast spectrum of activities? We recently proposed, in view of our recent results, that their apparent pleiotropic activities probably result of their ability to bind, chaperone and modulate the activity or half-life of many protein targets involved in crucial cellular regulations, such those involved in apoptotic cell death, tumorogenic and metastatic processes [21,23,24,25]. Small Hsps are characterized by their complex oligomeric structures allowing them to interact with each other to form homo and hetero-oligomeric structures of dynamic size (up to 700 kDa) [76,77,78]. Moreover, HspB1, HspB5 and HspB4 contain several serine sites that can be phosphorylated by specific kinases, including stress and MAP kinases. The phosphorylation and oligomeric organizations of these proteins are dynamic and deeply modified by changes in the cellular environment [15,21,37,77,79,80,81,82,83]. Indeed, these structural modifications are reversible and probably act as sensors of the cellular environment. For example, depending on the apoptotic inducer, HspB1 differently reorganizes its phosphorylation and oligomerization status suggesting that multiple strategies are set up by this protein to counteract apoptosis [15,21,51,77]. Changes in small Hsps structure can lead to at least 300 different stoichiometries to allow them to interact with putative client proteins [84]. Their dynamic structural plasticity would then favor the recognition of the more appropriated pro-cancerous client proteins, hence allowing the cancer cell to grow and disseminate. The clients of HspB1, HspB5 and HspB4 that could participate in the escape to death, growth, survival and aggressivity of cancer cells are presented below and in Figure 1.

### 3.1. Clients as Receptors and Growth Factors Stimulating Cell Growth

HspB1 and HspB5 have been reported to stimulate constitutive cell division by interacting with different cytoplasmic receptors, such as VEGF, FGF-2 and Her2, leading to an enhanced activity of the MAPK kinase/MEK/ERK pathway. However, the first report of the interaction of a small heat shock protein with a receptor concerned HspB1 being an estrogen receptor-beta (ERbeta) associated protein that could act as a co-repressor of estrogen signaling [85,86]. More recently, it was shown that, in human breast cancer cells, HspB1 increases Her-2/neu protein stability through its ability to chaperone this receptor. This resulted in an indirect negative effect of HspB1 towards Herceptin susceptibility [40]. Towards growth factors, one interesting small Hsps-client interaction has been detected during retinal angiogenesis. Indeed, in this tissue, HspB5 can chaperone vascular endothelial growth factor A (VEGF-A) and thus avoid its rapid degradation by the ubiquitin-proteasome machinery. The phenomenon results in a drastic stimulation of angiogenesis [87].

Abbreviations are the following: CD10: 100 kDa transmembrane metallo-endopeptidase; CD14: cluster of differentiation 14; TLR1: Toll-like receptor 1; TLR2: Toll-like receptor 2; TLR3: Toll-like receptor 3; NGF-beta: Nerve growth factor beta; Her2: Human Epidermal Growth Factor Receptor-2; ERbeta: Estrogen receptor beta; FGF-2: Fibroblast growth factor 2; VEGF: vascular endothelial growth factor; IKKbeta: inhibitor of nuclear factor kappa-B kinase subunit beta; DAXX: death domain-associated protein 6; PKC delta: protein kinase C delta; RhoA: Ras homolog gene family member A; PKCalpha: protein kinase C alpha; Akt: protein kinase B; P38: P38 mitogen-activated protein kinases; MK2: Mitogen-activated protein kinase-activated protein kinase 2 (MAPKAPK2); Phk: Phosphorylase kinase (PhK); PTEN: phosphatase and TENsin homolog; PRKD1: Serine/threonine-protein kinase D1; PPM1A: protein phosphatase 1A (formerly 2C), magnesium-dependent, alpha isoform; TRAF6: tumor necrosis factor receptor-associated factor 6; Raf, MEK, ERK: Mitogen activated protein (MAP) kinase pathways; Bax: Bcl-2 associated X protein; PEA-15: astrocytic phosphoprotein PEA-15; XIAP: X-linked inhibitor of apoptosis; p66Shc: 66 kDa isoform of ShcA (Src homology 2 domain containing transforming protein 2); G6PD: glucose 6-phosphate dehydrogenase; HDM2: Human double minute 2 homolog; IF: intermediate filaments; MAPs: microtubule-associated proteins; GFAP: Glial fibrillary acidic protein; GRIFIN: galectin-related interfiber protein; HDAC6: histone deacetylase 6; STAT2 and 3: signal transducer and activator of transcription 2 and 3; HSF-1: heat shock factor 1; GATA-1: globin transcription factor 1; Snail: zinc finger protein that binds and inhibits E-cadherin promoter to induce epithelial mesenchymal transformation; eIF4E: eukaryotic translation initiation factor 4E; eIF4G: eukaryotic translation initiation factor 4G; AUF1: AU-rich element (ARE)-binding protein; p90 ribosomal S6 kinase; Smad-Smurf2: Smad ubiquitination regulatory factor 2; p27kip1: cyclin-dependent kinase inhibitor p27kip1; Fbx4: Fbox only protein 4.

### 3.2. Clients Modulating Survival Signaling Pathways and Apoptosis

Apoptosis is a genetically programmed cell death negatively modulated by Hsps [7,38,147]. In contrast to the increased expression of Hsps by environmental insults, such as heat shock, no up-regulation of these proteins is observed in cells committed to apoptosis and only constitutively expressed pre-existing Hsps can be effective. This is particularly the case in human cancer cells where constitutively expressed small Hsps can counteract apoptotic process mediated by the immune system or therapeutic drugs. It is now believed that HspB1, HspB5 and HspB4 act by interacting with specific client proteins and modulate their activity in the initiation and execution phases of apoptosis [2,148].

**Figure 2:**
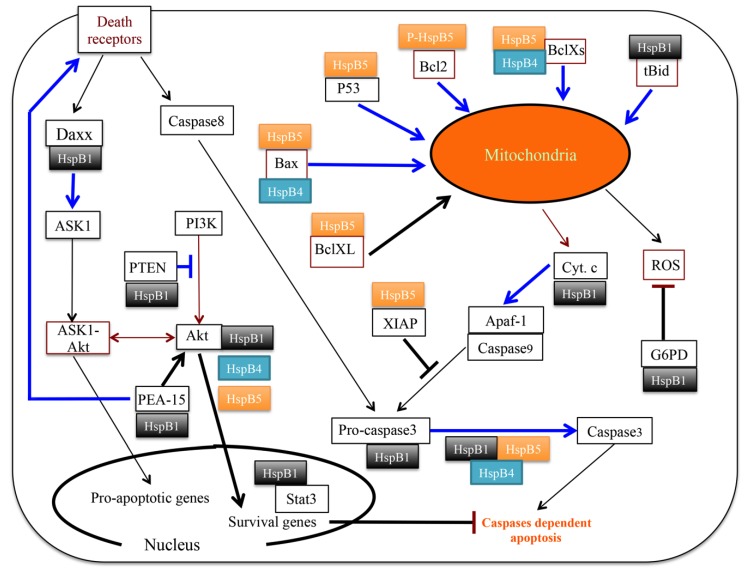
Cartoon that summarizes the apoptotic and survival pathways controlled by HspB1, HspB5 and HspB4. Large blue arrows are indicative of an inhibition of the corresponding pathways consequently of the binding of the clients to HspB1, HspB5 or HspB4. Black arrows or lines indicate that small Hsps either favorize the indicated pathways or just override them.

In cells committed to apoptosis, HspB1 is characterized by dynamic reorganization of its structural organization, particularly at the level of its phosphorylation and oligomerization status. These dynamic changes are signal transduction-dependent, hence favoring the hypothesis that this chaperone has multiple strategies, based on its interaction with specific targets, to counteract intrinsic and extrinsic apoptosis [51,77]. See Figure 2 for an illustration of these activities. Upstream of the intrinsic pathway, it was shown that by interfering with F-actin disruption and t-Bid translocation, HspB1 indirectly decreases the efficiency of the release of cytochrome c [149] and Smac-diablo [150] from mitochondria. Then, it interacts with cytosolic cytochrome c leading to impaired apoptosome and caspase-9 activation [108,151]. Downstream of these events, phosphorylated small oligomers of HspB1 interact with procaspase-3 and negatively regulate its activation [23,24,106]. It is also worth mentioning that the interaction of pro-caspase-3 with HspB1 counteracts the rapid degradation of this caspase by the ubiquitin-protasome machinery [23]. Hence, in addition of being able to inhibit a potent caspase effector, HspB1 has the surprizing property to protect it against a rapid proteolytic degradation. In the extrinsic pathways, HspB1 counteracts TNFαlpha, Fas and Trail induced apoptosis [7,11,152]. One well-defined target of HspB1 in the Fas pathway is Daxx [92,153]. Phosphorylated dimers of HspB1 interact with Daxx and indirectly prevent its association with both apoptotic signaling kinase1 (Ask1) and Fas; a phenomenon that blocks Daxx-mediated apoptosis. The cell survival kinase Akt is also a major target of HspB1 [95,154]. In fact, HspB1 is now considered as a key component of the Akt signaling cascade: Akt, BAD, mitogen-activated protein kinase kinase-3,6 and Forkhead transcription factors (Figure 2). By phosphorylating Bad, Akt abolishes its pro-apoptotic activity promoting cell survival. Another interesting protein recognized by HspB1 is the tumor suppressor PTEN that, by acting as a protein tyrosine phosphatase and a phosphatidylinositol phosphate (PIP) phosphatase, can negatively regulate the survival PI3K/Akt signaling pathway. In human breast cancer cells expressing high levels of HspB1, the interaction of PTEN with this chaperone further enhances the survival of these pathological cells [99]. In fact, it appears that HspB1 interaction with PTEN decreases the level of this phosphatase and therefore counteracts the PI3K-Akt survival pathway. Consequently, a geneticaly induced downregulation of HspB1 up-regulates PTEN level and blocks the survival pathway of cancer cells. This is an interesting example where HspB1 stimulates the degradation of a client protein instead of inhibiting its degradation. In gliomas, HspB1 was also described to participate in the signaling pathway that promotes cancer cell survival [155,156]. Another study showed that HspB1 silencing, which destroyed the interaction with PEA-15, coordinately inhibited proliferation and promoted Fas-induced apoptosis by regulating the PEA-15 molecular switch [13]. Hence, PEA-15 is an additional survival protein regulated by HspB1.

HspB5 and HspB4 are well-known anti-apoptotic polypeptides [7,157] whose major property is to negatively regulate the pro-apoptotic members of the Bcl-2 family, Bcl-X_L_ and Bax as well as caspase-3 [107]. HspB5 and HspB4 directly interact with caspase-3, Bax and Bcl-XS [107,110]. Moreover, in addition to interacting with these clients, HspB4 and HspB5 suppress their translocation from the cytosol into mitochondria and thereby prevent stress-induced apoptosis [110]. Similarly, HspB5 interacts with p53 to sequester its translocation to mitochondria and therefore indirectly inhibits its pro-apoptotic action towards anti-apoptotic Bcl-2 molecules [111] (see Figure 2). HspB5 was also found to abrogate calcium-activated Raf/MEK/ERK signaling pathway mediated p53-dependent apoptosis through inhibition of RAS activation [102]. In addition, both HspB4 and HspB5 could prevent UVA apoptosis through complex interactions with protein partners involved in regulating signaling PKCαlpha and Raf/MEK/ERK pathways [96]. Recently HspB5 was also reported to interact with XIAP, a most potent endogenous suppressor of apoptosis that acts by inhibiting caspases through a direct binding to these enzymes [113]. Thus, in spite of some common properties towards cell apoptosis, cell proliferation and tumor metastasis yet unknow molecular mechanisms have been described to explain the different role played by HspB4 and HspB5 in pancreatic carcinogenesis [54]. In that respect, it is important to recall that phosphorylation could be the culprit. One example supporting this point concerns serine 59 phosphorylation which shifts HspB5 activity from anti-apoptotic to pro-apoptotic since this phosphorylated isoform of HspB5 surprizingly sequesters Bcl-2 and blocks its translocation to mitochondria [158].

### 3.3. Senescence

In addition of protecting against programmed cell death, HspB1 can also counteract other host anti-cancer responses, such as the oncogene-induced senescence (OIS) pathway that acts as roadblock in cancer development but which is often lost upon cancer progression. The phenomenon which is p53 dependent has been detected by down-regulating HspB1 expression [10]. Indeed, depletion of HspB1 expression in cancer cells induces a senescent-like phenotype characterized by several morphological changes. Among them one can note a drastic multi-nucleation which results of the degradation of HDAC6, a specific client of HspB1 interacting with HspB1 oligomers phosphorylated at the level of serine 82 [23,24]. HDAC6 is a powerful modifier of carcinogenesis that contributes to oncogenic pathways activation [159]. A recent study performed in colorectal adenomas also suggests a possible role of HspB1 in overcoming PI3K/AKT induced OIS in tumours [160]. OIS depends on the activation of the p53 tumor suppressor protein pathway and induction of p21waf expression leading to cell cycle arrest [10]. Of interest, HspB1 interacts with HDM2, which serves as ubiquitin ligase (E3) to target p53 for degradation, and modulates its activity [10,161]. HspB1 down-regulation which leads to a senescent morphology appears therefore to result of the destabilization and degradation or inactivation of several client proteins involved in the senescent pathways, such as PI3K/AKT-PTEN, HDAC6 as well as the p53 stabilizator HDM2.

### 3.4. Clients Involved in Tumor Progression, Epithelial-to-Mesenchymal Transition, Metastasis

Additional to its role in protecting cancer cells by suppressing host anti-cancer response, such as apoptosis and senescence, other major pro-cancerous activities of HspB1 are linked to its tumorogenic property which promotes tumor growth and progression [9,14], invasion of tumor cells into surrounding tissues and dissemination to form metastatic colonies [17,18,19,28,29,162,163]. In spite of the fact that the molecular mechanism that drives HspB1 to promote tumor progression and metastasis is still not well understood, several different HspB1 clients have already been identified that participate in the achievement of these particular endeaviors. First, it is well known that HspB1 can directly modulate cytoskeleton integrity and indirectly extracellular matrix organization [23,78,117,118,164,165,166,167]. Indeed, recent observations clearly favor an important role played by HspB1 at the level of the cellular matrix suggesting that the relation between cancer cells and the normal cells that form the niche where the tumor grow is under the influence of small Hsps [19]. For example, HspB1 overexpression drives β-catenin from the cell membrane to the cytoplasm where both proteins interact with each other and consequently modulates cadherin–catenin cell adhesion proteins [131]. β-catenin also interacts with other proteins including heat shock transcription factor 1 (HSF1), P-cadherin, and caveolin-1. Disease-free survival were significantly shorter for patients with cytoplasmic expression of β-catenin consequently of the presence of HspB1. Hence, the interactions of β-catenin with HspB1 appears to play a key role in the molecular pathways that influence tumor cell survival. Another important role played by phosphorylated HspB1 in prostate cancer relates to the TGFbeta-induced activation of MMP-2 (matrix metalloproteinase type 2), an enzyme that digests components of the extracellular matrix surrounding tumor masses and subsequently stimulates human prostate cancer cells invasion [168]. TGFbeta-mediated increase in MMP-2 and cell invasion are inhibited in cells devoid of MAPKAPK2 or HspB1 expression as well as following the inhibition of HspB1 phosphorylation. Hence, both MAPKAPK2 and HspB1 appear necessary for TGFbeta-mediated cell invasion but the responsive clients of phosphorylated HspB1 acting in this pathway are still unknown. It was also shown that abrogation of 4T1 murine tumor migration induced by HspB1 down-regulation is associated with the repression of matrix metalloproteinase 9 expression together with a concomitant upregulation of its antagonist, tissue inhibitor metalloproteinase 1 [162]. This suggests that several metalloproteinases are under the control of HspB1. SPARC (secreted protein, acidic and rich in cysteine), a polypeptide playing an important role in cell-matrix interactions, is an additional polypeptide that uses HspB1 to modulate cell adhesion and migration. In colorectal cancer, stromal SPARC expression positively correlates with HspB1 expression and inversely with VEGF-A expression [169]. SPARC was also reported to promote invasion in gliomas through the up-regulation of the p38 MAPK/MAPKAPK2/HspB1 signaling pathway. This pathway promotes cancer cell survival by up-regulating the survival kinase pAKT activity through its interaction with HspB1 [155,156,170]. On the immunological side, high levels of HspB1 are associated with the repression of proteasome function and poor antigen presentation. By decreasing HspB1 level the proteasome activity is back to normal as well as the CD8+ T-cell-mediated tumor killing and the memory responses [171].

Back in 1997, a direct correlation between elevated level of HspB1 in the later stage of tumor progression and the invasive and metastatic potential of human breast cancer cells was confirmed *in vivo* in an assay measuring the number of lung metastases in mice injected with HspB1-transfected cells [17]. This study also reported that HspB1 overexpression decreases cell motility, but enhances invasiveness, adhesion, and growth in Matrigel. Consequently, suppression of HspB1 expression increases cell motility, but decreases *in vitro* invasiveness. Several recent studies have now confirmed that HspB1 is a key parameter in the emergence of metastasis [19]. A recent striking example is the promoting effect of HspB1 in breast cancer cells to metastasize and grow in the skeleton as bone tumours [12]. The epithelial-to-mesenchymal transition (EMT) is a fundamental parameter that controls metastasis formation, however the mechanisms regulating the phenomenon are still not well understood. In that respect, HspB1 participates in the maintenance of breast cancer stem cells through regulation of EMT and NF-κB transcription factor [31]. In prostate cancer, HspB1 also appears as an important player that promotes EMT since its depletion is associated with decreased cell migration, invasion, and MMP-2 activity [6]. The mechanisms involved in the promotion of the metastatic phenotype by HspB1 are not solved yet. It has been proposed, that HspB1 could modulate the expression of pro-metastatic genes [19], such as those dependent of the STAT3/Twist signaling [6]. Of interest, HspB1 and Twist expression are both elevated in high-grade prostate cancer tumors and HspB1 enhances the binding of the transcription factor STAT3 to the promoter of the Twist gene [6], an event that subsequently upregulates N-cadherin and downregulates E-cadherin expression, two hallmarks of EMT. However, how HspB1 mediates these effects is still not known (see below paragraph 3.7.1.).

HspB5 is well known for its involvment in cytoskeleton integrity [117,128]. Moreover, it also plays a role, at least in the kidney, for maintaining tissue integrity by interacting with Ksp-cadherin-16 and promoting the connection of this polypeptide to the cytoskeleton [132]. In cancer pathologies, HspB5 has been described to promote cell migration and invasion by modulating signal transduction pathways. For example, it induces EGF- and anchorage-independent growth of human breast basal-like tumors through the constitutive activation of the MAPK kinase/ERK (MEK/ERK) pathway [53]. Moreover, HspB5 can also act as an oncoprotein since, in nude mice, it has the intriguing property to transform immortalized human mammary epithelial cells in invasive mammary carcinomas that have the same aspect as basal-like breast tumors [53,172]. HspB5 expression in basal-like breast tumors is associated to poor patient survival independently of other prognostic markers. At least in pancreatic cancer cells, these properties are apparently not shared by HspB4 that acts, in spite of its protective property, as a negative regulator of carcinogenesis that retards cell migration and prevents tumor growth [73].

### 3.5. Clients on Cell Surface and Outside of Cells, Immunosurveillance, Angiogenesis and Autoantibodies

In spite of their major intracellular localization, recent reports mention that, similarly to the large Hsps such as Hsp70 [173], the small Hsps can localize in plasma membrane and be exported in the extracellular milieu [174,175,176]. Of interest, high levels of HspB1 cell surface expression were found to correlate with tumor growth and cell ability to metastasize [18]. Hence, do small Hsps, as the high molecular weight Hsps, associate with immunogenic peptides to bring an immune response to cancer cells [177] or are immunosupressive [178]? In that regard, an immunoregulatory activity has been associated to extracellular HspB1 that could contribute to immunopathology through the ability of this chaperone to interact with monocyte-derived dentritic cells and alter their activity, hence favoring immunosuppression. Extracellular HspB1 has also been described to act as a signaling molecule that activates NF-κB in macrophages [179]. In addition, a proangiogenic effect of HspB1 has been recently reported that depends on NF-κB activation [105]. Indeed, recombinant human HspB1 can recognize and interact with Toll-like receptor 3 (TLR3) at the surface of human microvascular endothelial cells (HMECs) grown as monolayers or spheroids and favors spheroid sprouting, NF-κB dependent VEGF gene transcription and promotes secretion of VEGF-activating VEGF receptor type 2 and angiogenesis. Hence, TLR3 can now be considered as a novel HspB1 interacting protein that indirectly promotes tumor growth.

An important point to keep in mind relates to the fact that circulating extracellular small Hsps can have pathology-dependent roles, similarly to their intracellular counterparts, that is they can either be deleterious or beneficial to patients. For example, circulating HspB1 is well known for its atheroprotective effect [176]. It also appears associated with micro- and macrovascular complications in type1 diabetic patients and considered as a novel marker for diabetic neuropathy [180]. The role of these extracellular proteins in normal physiological conditions will remain to be determined before therapeutic approaches are launched to target these circulating Hsps.

Another intriguing phenomenon concerns the presence of antibodies targeting HspB1 and HspB5 in the aqueous humor of normal tension glaucoma patients [181]. More surprizing is the fact that exogenously applied HspB1 antibody can penetrate in human retina neuronal cells and promote neuronal apoptosis by counteracting HspB1 anti-apoptotic activity [182]. This suggests that autoantibodies to small Hsps may impair cell survival, an activity that could be beneficial to cancer patients and be useful parameters in tumor diagnosis [26]. Circulating Hsps and anti-Hsps antibodies are now considered as being part of the recently defined “stress observation system” [173].

### 3.6. Clients Involved in Inflammation and in Controling Intracellular Redox State

Fledgling tumors are known to hijack inflammation and use it to accelerate the progression towards full-blown cancer [183]. Activation of NF-κB and a network of signaling molecules, which are key modulators in driving inflammatory cells to cancer cells, facilitate angiogenesis and promote the growth, invasion, and metastasis of tumors. In that respect, it is of great interest to note that HspB1 is essential for pro-inflammatory cell signaling and the expression of pro-inflammatory genes [184]. This protein is indeed required for both IL-1 and TNF-induced signaling pathways for which the most upstream common signaling protein is the pivotal kinase TAK1. Hence, downstream signalling by p38 MAPK, JNK and their activators (MKK-3, -4, -6, -7) and IKKbeta as well as expression of the pro-inflammatory mediators, cyclooxygenase-2, IL-6, and IL-8 are all dependent on HspB1. Unfortunately, the client(s) targeted by HspB1 are still unknown but should reside at the level or more upstream of TAK1. A surprizing finding has been made recently concerning HspB5 interaction, in the plasma of patient suffering of multiple sclerosis, with at least 70 different pro-inflammatory mediators (acute phase proteins, members of the complement cascade, and coagulation factors). Interaction with HspB5 reduces the concentration of these polypeptides leading to decreased inflammation [185]. Whether such a phenomenon occurs in patients suffering from cancer pathologies should urgently be tested. Another anti-inflammatory pathway linked to extracellular HspB5 concerns the activation of an immune-regulatory response in macrophages via endosomal/phagosomal CD14 and Toll-like receptors 1 and 2, two new HspB5 interacting proteins [104]. These reports clearly show that HspB1 and HspB5 act differently towards inflammation.

Inflammation is also linked to oxidative stress and, in this regard, expression of HspB1 or HspB5 is known for quite a while to induce a pro-reducing state in cells [78,83,115,152,186,187,188,189,190,191,192,193,194,195,196,197]. Indeed, decreased levels of intracellular reactive oxygen species and nitric oxide as well as iron uptake are observed in HspB1 and HspB5 expressing cells concomitantly to the upholding of mitochondrial membrane potential (∆Φm) and glutathione in its reducing form [115,152,186,187,194,198]. Consequently, protein oxidation, lipid peroxidation, DNA damages and cytoskeleton architecture disruption by oxidative stress are attenuated [186,189,196,199,200]. At least in the case of HspB1, these protective effects appear to result of the up-regulated activity of anti-oxidant enzymes [115,193], the major one being glucose 6-phosphate deshydrogenease (G6PD), a client of HspB1 [25,116], an enzyme that provides the reducing power of the cell consequently of its ability to transform NADP^+^ in NADPH+H^+^ [201], hence leading to oxidoresistance [202]. We recently showed that highly phosphorylated small homo-oligomers of HspB1 are responsive of the interaction of this chaperone with G6PD [25]. Consequently, drugs that interfere with the pro-reducing capability of HspB1 and HspB5 by interfering with their interaction with anti-oxidant enzymes may improve the killing efficiency of anti-cancer therapeutic drugs or conditions which depend on the intracellular redox state or the production of reactive oxygen species for their action, such as the Hsp90 inhibitor 17AAG or X-ray irradiation [196,203].

### 3.7. Clients Regulating Gene Expression

#### 3.7.1. Transcription

STAT3 is a crucial transcription factor whose persistant activation is involved in the maintenance and anti-apoptotic status of many cancerous cells through the activation of anti-apoptotic genes, such as the one encoding survivin [204,205,206,207]. Interaction between STAT3 and HspB1, reported already in 2004 [208], was confirmed recently [23,135]. STAT3 interacts with many different polypeptides and is transcriptionaly activated by tyrosine phosphorylation by either Jack2, c-Src, epidermial growth factor or other tyrosine kinases in response to various cytokines and growth factors [209,210]. It then forms homo- or hetero-dimers that translocate to the cell nucleus and bind to DNA. Of great interest, down-regulation of HspB1 expression decreases the level of phosphorylated STAT3 [23]. This suggests that the half-life of activated STAT3 is dramatically expanded through its interaction with HspB1. Hence, the transcriptional activation of STAT3, consequently of its phosphorylation and nuclear translocation, appears to require the presence of HspB1 [23]. Moreover, HspB1 has also been reported to be essential for the binding of STAT3 to the Twist promoter, hence promoting EMT and metastasis [6]. However, the role of HspB1 towards STAT3 is probably more complex than it has been originally thought since, depending upon the mutational background of the tumor, STAT3 can either be pro-oncogenic or have a tumor suppressor activity [211]. For example, in colorectal cancer cells STAT3 down-regulates Snail-1 expression levels and thus suppresses EMT and metastasis [212]. Hence, depending on the tumor, the relation between HspB1 and STAT3 may have different consequences. Whether HspB1 phosphorylation plays a role in this phenomenon will have to be tested. HspB1 also interacts with the transcription factor STAT2 and avoid its degradation by the ubiquitin proteasome pathway [23]. STAT2 is mainly involved in the control of anti-viral responses through its activation by interferon-γ [205]. STAT2 was shown to specifically interact with serine 78 and 82 phosphorylated HspB1 large oligomers [23,24]. HspB1 also plays a role towards GATA1, a transcription factor essential for erythroid differentiation, which is heavily mutated in almost all megakaryoblastic leukemias in patients with Down syndrome [213]. HspB1 interacts with GATA1 and down-regulates its content and activity by inducing its ubiquitination and proteasomal degradation during terminal erythroid differentiation [137]. The role played by HspB1 towards GATA1 in these pathologies will have to be determined. An additional target of HspB1 is HSF1 (heat shock factor 1), the transcription factor responsible for Hsps expression that plays an important role in tumorogenesis [4]. In that regard, HspB1 is involved in SUMO-2/3 modification of HSF1 [136]. In the nucleus, large oligomers of HspB1 interact with HSF1 and induce its modification by SUMO-2/3 on lysine 298. This modication results of the interaction of HspB1 with the SUMO-E2-conjugating enzyme Ubc9 and takes place downstream of HSF1 phosphorylation. This modification does not affect HSF1 DNA-binding ability but blocks its transactivation function. Future studies will have to test whether SUMO-2/3 favors HSF1 activity as a signal modulator stimulating kinase activity, regulating energy metabolism and permitting the development of polyploidy in cancer cells or as an inhibitor of transcription which, in cooperation with NuRD family factors, can repress genes that oppose metastasis [4].

HspB5 has not yet been described to play a role in transcriptional modifications favoring the development of cancer cells. In contrast, HspB4, which can act as an inhibitor of tumorogenesis, has been proposed to halt cell transformation and retard cell migration by modulating the expression and activity of the transcription factor AP-1 [54].

#### 3.7.2. Translation

HspB1 is well known for its activity as an inhibitor of translation during heat shock that limits the accumulation of unfolded proteins. In heat shock treated cells, HspB1 interacts with the initiation factor eIF4G, but not with the other factors of the cap-binding initiation complex such as eIF4E, eIF4A, eIF4E kinase Mnk1 and poly (A)-binding protein [138]. HspB1-eIF4G interaction facilitates the dissociation of cap-initiation complexes and promotes translation inhibition. In cancer cells, active translation machinery is a prerequisite for cell growth and proliferation and once again HspB1 plays a major role in the protein translational initiation process. A study performed in prostate cancer showed that HspB1 up-regulation, which is particularly intense after androgen withdrawal, confers broad-spectrum treatment resistance including chemotherapy. Analysis of the enhanced cell survival during cell stress induced by castration or chemotherapy resistance revealed that it resulted mainly of HspB1 protection of the protein synthesis initiation process. In that context, HspB1 was found to interact with the translation initiation factor eIF4E [139]. Moreover, HspB1 downregulation decreased eIF4E expression at the protein, but not mRNA, level. The phenomenon clearly shows that HspB1 acts as a chaperone that stabilizes eIF4E half-life by counteracting its degradation by the ubiquitin-proteasome pathway. Hence, by protecting translational initiation HspB1 can indirectly enhance cancer cell survival through an efficient translation of mRNAs encoding polypeptides. No activities of HspB5 or HspB4 have yet been detected in that particular field of investigation.

## 4. Peptides, Natural Molecules and Drugs that Inhibit the Proliferation of Human Cancer Cells through Their Ability to Interact with Small Hsps, Therapeutic Approaches

Through their ability to interact with each other small Hsps form complex homo- and hetero-oligomeric structures that deeply modify the activity of the parental molecules. For example, it has been observed that some exogenous mutants of HspB1 can be dominant negative knocking out the activity of endogenous wild-type HspB1 through formation of inactivated hetero-oligomeric complexes that cannot recognize protein partners. In that respect, the substitution of the only cysteine residue in HspB1 sequence by an alanine is very efficient [108,214]. We also recently reported the drastic dominant negative effect mediated by the R120G mutant of HspB5 towards HspB1 [78]. Hence, a mutated small Hsp can alter the protective activity of other members of this family of chaperones expressed in the same cell. This probably occurs through a modifcation of their ability to recognize specific client polypeptides [25,78]. In spite of these observations, the use of mutants as therapeutic approaches to modulate the activity of some small Hsps is not a realistic therapeutic approach. On the contrary, the use of peptides derived from crucial domains that mimick their structural organization and chaperone activity is more reasonable. Only few examples have been reported in the litterature about the effect of peptides mimicking HspB1 and HspB5 activities. One example concerns the interaction of HspB1 with seven amino acids of the PKC delta-V5 heptapeptide region (residues 668 to 674, E-F-Q-F-L-D-I) of Protein Kinase C delta (PKCdelta). This PKC region can sensitize human cancer cells by sequestring HspB1, hence inhibiting its interaction with pathological protein partners [93,200]. This resulted in the inhibition of HspB1-mediated resistance against drugs, such as cisplatin and DNA damaging agents. On the other hand, peptides can stimulate small Hsps activity such as in the case of HspB5 where peptides derived from crucial domains of this chaperone mimicked its positive action against the fibrillation of amyloidogenic proteins [215]. The search for peptides that, instead of mimicking small Hsps, directly modulate their interactions with client proteins is another approach that can lead to the discovery of potent peptidomimetic drugs. Peptide aptamers belong to this second class of peptides [16,216,217,218,219,220]. In that regard, we have described HspB1 interacting peptide aptamers that could either down-regulate or stimulate the anti-apoptotic and cytoprotective activities of this stress protein, hence confirming the importance of the structural organization of this chaperone [16,221]. Moreover, *in vivo* head and neck tumor xenograft studies revealed that aptamers could reduce tumor growth more efficiently than HspB1 depletion [16]. Aptamer poisoning altered HspB1 structural organization and probably its abberant pro-cancerous interactome. These observations support the hypothesis that, in addition of drugs, anti-senses or RNAis strategies that decrease HspB1 levels [6,13,222], therapeutic chemical drugs could be designed to directly destabilize small Hsps oligomeric structure and their pro-cancerous interactome networks. 

Drugs that decrease HspB1 expression are well known but usually poorly specific. One can cite quercitin [223], berberin derivatives (EPO Patent 0813872-A1), paclitaxel [224], KNK437 [225] and interferon-γ which suppresses HspB1 basal transcription and promoter activity thus enhancing hyperthermia-induced tumor cell death *in vitro* and tumor suppression *in vivo* [226,227]. Far more interesting drugs are those that directly interact with HspB1 and modulate its activity, here are some of them:

(1) Biphenyl isooxazoles such as 5-(5-ethyl-2-hydroxy-4-methoxyphenyl)-4-(4-methoxyphenyl) isoxazole (KRIBB3) inhibit tumor cell migration and invasion by blocking protein kinase C-dependent phosphorylation of HspB1 through their direct binding to HspB1 [228].
(2) RP101 (*E*)-5-(2-bromovinyl)-deoxyuridine, BVDU, brivudine), a 2'-deoxyuridine derivative (thymidine analogue). This well-known anti-viral drug, particularly efficient againt Herpes simplex virus (HSV-1) and varicella zoster virus (VZV), was recently reported to drastically enhance the efficiency of human pancreatic cancer chemotherapy. RP101 acts by inhibiting HspB1 interaction with pro-cancerous binding partners, such as cytochrome c, pro-caspase-3, and Akt1 [229]. This results in a stimulation of the activity of caspase-9 and apoptosis efficiency. In addition of being incorporated into viral DNAs and inhibiting the action of DNA polymerase and viral replication, RP101 can bind to a specific site of the *N*-terminal domain of HspB1 characterized by two phenylalanine residues (Phe29 and Phe33). This probably interferes with the dynamic oligomerization of this chaperone and its ability to recognize client proteins. In addition of being approved as an anti-viral drug, RP101 efficiency towards pancreatic cancer patients is actually in clinical phase II/III. Of interest, an increased efficiency has recently been reported for novel HspB1-targetting derivatives of RP101 [230].
(3) Clerodane diterpenoids form a class of terpene derivatives biosynthesized in plants, particularly those of the Lamiaceae family. Among them, neo-clerodane diterpenoids from *Salvia* spp. were recently described as compounds inhibiting the proliferation of several human cancer cell lines. Chemical proteomics approach analyzing the cellular interactome of a representative clerodane diterpenoid molecule, hardwickiic acid (HAA), revealed HspB1 as a major HAA interacting protein partner in U937 leukemic cells [231]. The interaction was confirmed by several other approaches, including surface plasmon resonance measurements, limited proteolysis, and biochemical assays. Hence, the antitumor potential of clerodane diterpenoids may be linked to the inhibition HspB1 oncogenic properties. Moreover, this observation may be crucial for the future discovery of potent HspB1 diterpenoid-based chemical inhibitors.

Analyzing crucial modifications in the structure of small Hsps that play a role in cancer pathologies is another approach that could lead to the discovery of new drugs. In that respect, argpyrimidine modification could be of interest since this modification decreases HspB1 ability to bind cytochrome c and consequently alters its anti-apoptotic property [109]. The phenomenon is probably linked to the important structural role played by arginine residues in the conserved α-crystallin domain of small Hsps [76,78,232,233].

## 5. Conclusions

Additional to their up-regulated levels and broad molecular chaperone activities in stress conditions, small Hsps are now described to have an incredible number of fundamental functions in unstressed human cells [5,27,83,234]. The phenomenon is particularly intense in pathological cells where they show an apparent high level of constitutive expression. These multiple cellular functions result of the fundamental property of Hsps, first discovered in stressed cells, which consists in their interaction with client polypeptides to control folding. This activity can have several consequences: if the folding of the clients is not adapted to cellular conditions, small Hsps can participate in their refolding or degradation. Small Hsps can also artificially maintain their half-life by permanently interacting with them. The folding property of small Hsps can also modulate the activity of enzymes and/or trigger specific modifications to better adapt them to changes in cell physiology. Hence, the polypeptides that are controled by small Hsps could have crucial functions, particularly in pathological cells. For example, the protective role of small Hsps towards some clients could be beneficial against cell degeneration [22]. In contrast, other clients can be deleterious consequently of their ability to help cells to evade death and proliferate, such as cancer cells. In vew of all these observations, it was concluded that small Hsps are therapeutic targets. However, they are tricky ones since, depending on the pathology, their activities will have to be either stimulated or abolished.

The major drawback to understand the chaperone functions of small Hsps is the still not well characterized role played by their highly complex and dynamic combinatorial phospho-oligomeric structures that allow them to interact with a specific polypeptide in a define cellular condition. Despite this unsolved problem, the number of proteins that are nowdays discovered to interact with small Hsps is growing exponentially. Here, we have analyzed the putative pro-cancerous role of the major polypeptides that interact with HspB1, HspB5 and HspB4. It is of particular interest to note that these proteins are mainly engaged in processes that drastically modulate the development of tumors and their metastatic dissemination. For example, they can promote constitutive cell division by interacting with cytoplasmic receptors (VEGF, FGF-2, Her2) leading to enhanced activity of MAPK kinase/MEK/ERK pathway. HspB1, HspB5 and HspB4 are also particularly effective in inhibiting extrinsic and intrinsic apoptosis by using their own specific strategies. In that respect, they knock down key pro-apoptotic factors (cytochrome c, caspase-3, pro-apoptotic Bax, BclXs, tBid, P53, DAXX) and stimulate or override the anti-apoptotic activity of other factors (PEA-15, XIAP). Another important phenomenon highly stimulated by HspB1 is the survival pathway while the oncogene induced senescence (OIS) pathway appears inhibited. In that respect, the major targets are PTEN, Akt and the transcription factor STAT3 that induces expression of anti-apoptotic genes and promotes survival through cross talk with the Wnt/beta-catenin pathway [204,235]. Finally, HspB1 and HspB5 have been described to promote tumor progression, EMT and metastasis. In that regard, small Hsps are well-known regulators of the cytoskeleton and cellular matrix organization. Other crucial targets are SPARC, matrix metalloproteinase type 2 and β-catenin as well as still unknown ones involved in the up-regulation of pro-metastatic genes through STAT3/Twist signaling. Recently, reports have mentioned that tumor growth and cell ability to metastasize correlated with a fraction of the cellular content of HspB1 being localized in the plasma membrane and extracellular milieu, leading to a possible immunoregulatory or signaling activity such as the proangiogenic effect recently described to be associated to this circulating protein. Many other polypeptides are targeted, including modulators of inflammation, transcription and translation factors, regulators of protein stability and anti-oxidant enzymes, that all can have an impact on the development and maintenance of tumors. Hence, these studies lead to the apparent view that small Hsps interactomes could be controled by some cancer cells to promote their development. Further analysis of the phosphorylation patterns of HspB1, HspB5 and HspB4 will be required to test whether these modifications modulate their ability to negatively or positively modulate the pro-cancerous activity of their clients. Another parameter that generates even more complexity is the fact that, when expressed in the same cells, small Hsps can interact [236,237] and form multiple combinatorial chimeric hetero-oligomeric complexes that are differentially phosphorylated, such as HspB1 and HspB5 which are highly prone to interact [25,78]. Nothing is known concerning the interactomes of these chimeric structures that seem to have lost the properties associated to the parental homo-oligomers. Hence, these examples clearly illustrate the complexity associated to the interaction of small Hsps with defined polypeptides targets. More in-depth structural studies are urgently needed before comprehensive interactomes could be elaborated and used to search for specific therapeutic anti-cancer drugs.
